# Health Seeking Behaviour and Utilization of Health Facilities for Schistosomiasis-Related Symptoms in Ghana

**DOI:** 10.1371/journal.pntd.0000867

**Published:** 2010-11-02

**Authors:** Anthony Danso-Appiah, Wilma A. Stolk, Kwabena M. Bosompem, Joseph Otchere, Caspar W. N. Looman, J. Dik F. Habbema, Sake J. de Vlas

**Affiliations:** 1 Department of Public Health, Erasmus MC, University Medical Center Rotterdam, Rotterdam, The Netherlands; 2 Centre for Reviews and Dissemination, University of York, York, United Kingdom; 3 Parasitology Department, Noguchi Memorial Institute for Medical Research, Legon, Ghana; Centre Suisse de Recherches Scientifiques, Côte d'Ivoire

## Abstract

**Background:**

Schistosomiasis causes long-term illness and significant economic burden. Morbidity control through integration within existing health care delivery systems is considered a potentially sustainable and cost-effective approach, but there is paucity of information about health-seeking behaviour.

**Methods:**

A questionnaire-based study involving 2,002 subjects was conducted in three regions of Ghana to investigate health-seeking behaviour and utilization of health facilities for symptoms related to urinary (blood in urine and painful urination) and intestinal schistosomiasis (diarrhea, blood in stool, swollen abdomen and abdominal pain). Fever (for malaria) was included for comparison.

**Results:**

Only 40% of patients with urinary symptoms sought care compared to >70% with intestinal symptoms and >90% with fever. Overall, about 20% of schistosomiasis-related symptoms were reported to a health facility (hospital or clinic), compared to about 30% for fever. Allopathic self-medication was commonly practiced as alternative action. Health-care seeking was relatively lower for patients with chronic symptoms, but if they took action, they were more likely to visit a health facility. In a multivariate logistic regression analysis, perceived severity was the main predictor for seeking health care or visiting a health facility. Age, socio-economic status, somebody else paying for health care, and time for hospital visit occasionally showed a significant impact, but no clear trend. The effect of geographic location was less marked, although people in the central region, and to a lesser extent the north, were usually less inclined to seek health care than people in the south. Perceived quality of health facility did not demonstrate impact.

**Conclusion:**

Perceived severity of the disease is the most important determinant of seeking health care or visiting a health facility in Ghana. Schistosomiasis control by passive case-finding within the regular health care delivery looks promising, but the number not visiting a health facility is large and calls for supplementary control options.

## Author Summary

The World Health Organization recommends that long-term benefit of schistosomiasis control should include treatment in local health facilities. This means that patients should visit a hospital or clinic with their complaints. However, little is known about whether they do so. We conducted a study in three regions of Ghana and interviewed two thousand people about whether they recently had schistosomiasis-related symptoms such as blood in urine or blood in faeces, and what they had done about it. We included fever (mostly caused by malaria) for comparison. We found that 40% of patients with urinary symptoms sought care compared to 70% of those with intestinal symptoms and 90% with fever. Overall, only 20% of all schistosomiasis-related symptoms were reported to a hospital or clinic, compared to 30% for fever. Self-medication with allopathic (i.e., orthodox) medicines was the main alternative. Our study showed that the most important determinant for seeking health care or visiting a health facility is perceived severity of the symptom. Factors such as age, sex, socio-economic status and geographic region showed no impact or a clear pattern. We conclude that many schistosomiasis patients do not visit a health facility, the only place with effective drugs, necessitating additional control measures.

## Introduction

Schistosomiasis leads to chronic ill health and significant economic burden [1],[2],[3],[4]. *Schistosoma haematobium* and *S. mansoni* are widespread in Africa causing urinary and intestinal schistosomiasis, respectively. Both species are found in Ghana, sometimes as mixed infection in the same person. For most people who are repeatedly exposed, the severity of disease depends upon the intensity of infection. Haematuria (blood in urine) and dysuria (painful urination) are the main early symptoms of urinary schistosomiasis and diarrhoea, blood in stool and abdominal pain for intestinal schistosomiasis. Over 70% of infected children show one or more early symptoms and signs of disease [5],[6]. Adults, who usually have light infections, are often asymptomatic but some develop late pathology after prolonged infection.

After the introduction of praziquantel in the late 1970s, the World Health Organization (WHO) in association with ministries of health of several endemic countries met in 1983 to assess viable control strategies. The high price of praziquantel in those days led to the endorsement of morbidity control with emphasis on treating individuals with early symptoms of the disease or having high egg counts [7]. As the development of pathology and disease are closely associated with intensity and duration of infection, the assumption was that treating these cases will prevent most late-stage complications which are insidious and occur many years after the infection. This control strategy was reviewed by the WHO Expert Committee in 1991 and proved to be effective in vertical control programmes, but sustainability was identified as a major problem due to the over-reliance on foreign donors [8]. To be sustainable, there should be a prominent role of the existing health care delivery system that builds on local structures and capacity, called a horizontal approach [9],[10].

With the highly reduced price of praziquantel, the regular health care system may now be able to procure praziquantel for the treatment of schistosomiasis patients visiting health facilities. To understand what policy changes are necessary for successful integrated control, it is important to first know the extent to which currently available services contributes to morbidity control via passive case finding. This critically depends on the community's perception of the disease, health seeking behaviour of individuals and socio-economic factors [11]. For seeking health care, people must consider the symptoms a health threat and have resources available [12]. Health care seeking behaviour is not only a matter of knowledge about the cause and treatment of the disease, but also of perceived seriousness and duration, cultural practices and socio-economic status [13]. Perceived quality of the health care expected, availability and cost of medicine, distance to hospital, and user fees charged also influence hospital visit [14].

Paucity of information about health care seeking behaviour for schistosomiasis symptoms led us to conduct our previous study [15]. The results showed that around 70% with blood in urine or painful urination did not seek health care. Symptoms associated with intestinal schistosomiasis (diarrhoea, blood in stool and abdominal pain) and also fever usually led to health care although self-medication with allopathic drugs was commonly practiced. On average 20% of schistosomiasis-related signs and symptoms were reported to health facilities. Teenagers consistently showed lower tendency to take action for their symptoms than children under 10 years and adults. Socio-economic status and duration of symptoms did not appear to influence health-seeking behaviour, and lack of money (43%) and symptom perceived as not serious enough (41%) were the commonest reasons for not visiting a clinic.

We conducted a larger study in multiple locations in Ghana to learn whether patterns of health seeking behaviour are consistent across other parts of the country, get better insight into regional characteristics, and to enable formal analysis of determinants of action. The study also investigated the effect of new factors namely, geographic location, distance and perceived quality of health facility. We discuss implications for morbidity control of schistosomiasis.

## Materials and methods

### Ethics Statement

The study protocol was reviewed and approved by the Institutional Review Board (IRB) of the Noguchi Memorial Institute for Medical Research (Accra) and Erasmus MC (Rotterdam). Thereafter, a detailed plan of the study and its objectives were submitted to the Ethical Committee of the Ministry of Health (Ghana) for approval, which was granted. Copies of approved ethical clearance were sent to all concerned parties and authorities including District Chief Administrators and Directors of Health Service. The purpose of the study and its objectives were explained to local authorities, opinion leaders and community members at a durbar. Verbal informed consent to participate in the study and household interview was obtained from heads of households, each individual and parents of minors according to the principles of the declaration of Helsinki. Written consent was not sought because of high levels of illiteracy of the study population. The IRB approved the use of oral consent prior to the start of the study. Subjects were assured about confidentiality of information obtained from them and personal identifiers were removed from the dataset prior to analysis. Names of participants were recorded in interview books and boxes to document consent were ticked when consent was given, a cross otherwise. We did not encounter problems or individuals who did not want to participate in the study.

### Health care delivery in Ghana

Health care delivery in Ghana is based on the Primary Health Care concept [16]. At least one government-owned hospital is located in each district capital and staffed with one or more qualified medical doctors, nurses, pharmacists, laboratory technicians, auxillary nurses and other support personnel. The district hospitals deal with all cases except specialised care, and serious cases are referred to the regional tertiary hospitals. There are also a number of health centres mostly without laboratory facilities in the sub-districts which are manned by a medical assistant or a nurse. There are also private clinics and chemical shops/pharmacies in the district and sub-district capitals. In the health facilities in our study sites treatment for schistosomiasis-related symptoms was mostly based on signs and symptoms (presumptive treatment) and the system of payment during the period of the study was *cash and carry* (out of pocket payment) where a patient was required to make full payment for consultation before treatment was provided. Laboratory tests, if indicated, were referred. Within the cash and carry system, essential drugs were mostly kept in the health facilities for purchase, but patients had to obtain other drugs from private pharmacies.

### Selection of study villages

This questionnaire-based study was conducted in 2002–2003 in three different sites of the country (south, central and north), which reflect Ghana's ethnic and cultural diversity and distribution of general infrastructure that may influence health seeking behaviour.

The main criteria for selecting a village for inclusion in the study were; 1) the village must be endemic for either urinary or intestinal schistosomiasis or both, 2) there must be a health facility where at least 80% of the inhabitants would visit when ill or experiencing minor ailments, and 3) the village should have a stable population where new entrants moving into or old inhabitants leaving the population is minimal. From the south we selected two villages, relatively near and far from health facilities utilized by both villages to test the impact of distance on health seeking behaviour.

One village, Biu, along the Tono irrigation site was selected from the north dry semi-desert part of the country: Biu had about 1200 inhabitants and prevalence of 56% and 48% for intestinal and urinary schistosomiasis, respectively. There is one government health centre in Biu and the referral hospital is located in the district capital (Navrongo) about 20 km away. There are no chemical shops in Biu and patients including schistosomiasis-related cases obtain most of their drugs prescribed to them from private drug stores in Navrongo. The main occupation of adults aged over 18 years is farming on the irrigation fields, but some also rear cattle and sheep. The vast majority of adult females engage in petty trading but a reasonable number also farm. The Tono Lake formed as a result of the dam is used for drinking, bathing, domestic activities, fishing and farming thereby making the lake the main source of the infection. Amankwah *et al*. [17] provides a detailed description of the Biu study area.

From the Ashanti region in the central tropical forest of Ghana, two adjacent villages endemic for only urinary schistosomiasis were selected: Kyereyase (about 1200 inhabitants and prevalence >71%) and Nyamebekyere (about 300 inhabitants and prevalence >65%). The demography and epidemiology of these two villages were so similar that we considered them as one in our analysis. There was no health facility or chemical shop in these villages, but outreach services delivered by public health nurses from the district hospital were offered in Kyereyase. The nearest health facility utilized by some patients from the two villages was a private clinic located in Nerebehi about 3 km from Kyereyase and 6 km from Nyamebekyere. The district hospital located in Nkawie (the district capital) about 5 km from Kyereyase also serves as the referral centre for cases from most private clinics and health centres in the surrounding towns and villages. Some schistosomiasis-related cases from Kyereyase and Nyamebekyere are reported to health facilities in the regional capital in Kumasi about 7 km away. The two study villages are separated by the River Offin, which is the main source of water for drinking, bathing and domestic activities. Children swim in the river on regular basis making the river the main source of the infection. Kyereyase is accessible by road but the inhabitants of Nyamebekyere live behind the river and have to walk through the forest to cross River Offin midway by a canoe in order to join a vehicle from Kyereyase to nearby commercial towns or health facilities. Cocoa and rice are the main cash crops cultivated by the farmers in these villages.

Two villages were selected from the Greater Accra region in the south coastal savannah belt, which are endemic for both urinary and intestinal schistosomiasis: Manheim (about 3000 inhabitants and prevalence (60% and 56%) for intestinal and urinary schistosomiasis, respectively and Tomefa (about 1500 inhabitants and prevalences of 72% and 70%). Manheim had two private clinics where some schistosomiasis-related cases reported for treatment and one chemical shop where individuals bought their drugs. Tomefa is about 10 km from the nearest health facility and has no chemical shop. The two villages utilize the same health facilities in Manheim and Kasoa, a nearby commercial centre about 1 km from Manheim. There are two public health centres and about ten private clinics in Kasoa, but most people from Manheim and Tomefa with minor ailments including schistosomiasis-related symptoms utilize the government-owned health facilities. These health facilities mostly refer cases to the nearest government-owned polyclinics in Accra, about 10 km away. The inhabitants of Tomefa visit the health facilities in Manheim and Kasoa either by a car or canoe. The distance is variable depending on the medium of transport used: about 5 km by a canoe and 10 km by car. However, boats and canoes are rarely used as means of transport by non-fishermen including women and children because of the dangers associated with it. The main occupation of adult men in these villages is fishing whilst the women are mostly petty traders. The inhabitants of these villages fetch water from the lake for drinking, bathing and domestic activities whilst children swim in it on regular basis making the lake the main source of schistosomiasis infection. Manheim and Tomefa are located in the same area as our earlier study along the Densu Lake [15].

### Population sampling and data collection

We set out to interview at least a quarter of the population in each village, resulting in a total sample size of about 1800 individuals. This sample was largely based on practical feasibility. Based on our earlier study, we expected this number to be sufficient for meaningful statistical analysis. For the sampling, each study village was mapped, divided into sectors based on topography and closeness to the source of infection, houses numbered and each individual registered according to household. Households were randomly selected using computer generated random numbers. The questionnaire used in our earlier study [15] was slightly adapted and administered to all inhabitants in the selected households. Each subject was assigned an identification code defining the village, sector, house number and serial number.

The questionnaire was made up of a section on date of interview, name of interviewer, start and end time of interview, questionnaire book number, house number, individual serial number and identification code; a demographic section including name, sex and age of subjects; decision-making indicators such as who requests health care, who provides money for health care, who accompanies the patient when visiting the hospital; socio-economic indicators such as level of education, occupation and property owned; indicators of schistosomiasis-related signs and symptoms such as symptom currently present or present during the past one week or one month, duration and severity; action taken for each symptom and the name of health facility last visited, perceived quality of care received and the length of time spent (including travel, waiting and consultation) when they visited this health facility. Symptoms were categorized as acute when present for up to one week, and chronic otherwise. The three severity classes mild, moderate and severe were based on self-reporting.

The signs and symptoms covered by the questionnaire were: blood in urine and painful urination (for urinary schistosomiasis), and blood in stool/bloody diarrhoea, diarrhoea, abdominal pain and swollen abdomen (for intestinal schistosomiasis). These signs and symptoms are only suggestive, but not necessarily, due to schistosomiasis. Fever (for malaria) was added as a serious and debilitating disease for comparison. Questions relating to signs and symptoms were asked in a random order. The socio-economic status of individuals was assigned by using the status of the household to which they belong. Three possessions (car, fridge and television) were selected as indicators of relatively high socio-economic status of a household. Any household owning one or more of these was considered as high socio-economic class, otherwise low.

Registered individuals from the randomly selected households in each village were interviewed, with parents or guardians answering for children aged under six years. Potential respondents for individuals who could not answer the questions for themselves, mostly children, were preferentially ranked as follows: mother, father, guardian (aunt, uncle or other close relatives). Registered individuals who were not present during the interview and subsequent follow-up visits were classified as permanently missing and excluded from the study. To minimize variability, the questionnaire, which took an average of 20 minutes each to administer, was applied by two trained interviewers who also did the interviews for our earlier study [15].

### Data management and statistical analysis

The data were double entered. The first entry was done manually using Epi-Info (version 6.04), and the second electronically using an Electronic Scanning Data Entry Machine. The two resulting datasets were compared and cleaned using Excel. In case of discrepancies, the hard copies were consulted and the necessary corrections made. Copies of the questionnaire books and back-ups of the electronic data were kept at the Noguchi Memorial Institute for Medical Research in Ghana and the Department of Public Health, Erasmus MC, Rotterdam. Descriptive and logistic regression analyses were done using SPSS version 15.0.

Using logistic regression we tested the impact of various factors on the tendency to take any action (i.e. to self-medicate or visit a health care facility) for each symptom separately, and on the tendency to visit a health facility (hospital or clinic) as first action given any action. First, univariate analyses were conducted for the following factors and interaction terms: age, sex, location, socio-economic status (SES), perceived severity and duration of symptom, whether ‘self’ or ‘other’ provided money for health care, perceived quality of health care, time usually spent in obtaining care from a health facility and the interaction terms- age×sex and severity×duration of symptom. Only these two interaction terms seemed relevant and sometimes tested significant in univariate analysis. The impact of distance to health facility was tested as part of location using the comparison between Tomefa (far) and Manheim (near).

Subsequently, we conducted multivariate analyses to obtain adjusted ORs. For each symptom, all variables showing an overall p<0.20 in the univariate analysis were selected for inclusion in the multivariate analysis as recommended by Hosmer and Lemeshow [18]. No further backward or forward selection procedures were conducted, but interaction terms were removed, because their effects were negligible in the multivariate analyses.

In subsequent steps, we grouped all symptoms together and re-analyzed the factors that influenced taking any action or visiting the hospital as first action. For this purpose, we did a multi-level analysis with bootstrapping, including a random factor component to account for repeated observations from the same individuals. These analyses resulted in significant impact of the interaction of various key factors with type of symptom, indicating it was not appropriate to combine different symptoms and analyse as one. Therefore, we report results for each symptom separately.

### Results

In total 2,002 individuals were interviewed, coming from 52 households (with an average of 6 members) from north (Biu), 75 (13) from central (Kyereyase/Nyamebekyere), 70 (8) from south (Tomefa), and 81 (8) from south (Manheim). There were 1010 females and 992 males of all ages, of which 614 were children under 10 years, 466 teenagers and 922 adults. Educational level among individuals aged over 15 years was low, 858 (77%) had no or only elementary education and<5% had attended a senior high school or had tertiary education, with the rest having up to junior high school (slightly above elementary school). The vast majority of adult men were farmers, fishermen or low-income earners, whilst most of the women were farmers, petty traders or both.

Age and level of education did not differ markedly between the different study sites (Table 1). However, sex and socio-economic status showed marked differences. The percentage of males in Tomefa (58%) differed from the other villages (42–50%), whilst relatively high socio-economic status was rare in the north (5%) compared to central (21%) and south (47–57%). The decision making process for obtaining health care did not differ between locations. The proportion of patients reporting to take more than half a day in visiting a health facility is much lower in the north than in other regions (34% vs. 64–98%). When asked what they would do if they had blood in urine, diarrhoea, blood in stool or fever over 96% reported they would seek health care.

**Table 1:** Characteristics of the study populations and indicators of health seeking behaviour.

Factor	North	Central	South (Tomefa)	South (Manheim)
Subjects interviewed	300	510	525	667
*Prevalence of infection*				
% *Schistosoma mansoni*	56	-	72	60
% *S. haematobium*	48	71	70	56
*Demographic characteristics*				
Males (%)	125 (41.7)	254 (49.8)	302 (57.5)	311 (46.6)
Children<15 years (%)	126 (42.0)	247 (48.4)	215 (41.0)	295 (44.2)
High education (%)*	5 (1.7)	10 (2.0)	19 (3.6)	24 (3.6)
High SES (%)#	16 (5.3)	106 (20.8)	246 (46.9)	380 (57.0)
*Decision making process to seek health care*				
Someone else requesting for health care	121 (40.3)	271 (53.1)	290 (55.2)	367 (55.0)
Someone else paying for health care (%)	100 (33.3)	130 (25.5)	170 (32.4)	223 (33.4)
*Health facility indicators*				
Perceived HF as good (%)†	244 (95.7)	339 (92.6)	232 (69.3)	349 (80.4)
≥ ½ a day for visiting HF (%)	77 (34.5)	269 (97.8)	190 (79.2)	214 (64.3)
*Tendency to take action* ‡				
Blood in urine (%)	294 (98.0)	500 (98.0)	504 (96.0)	642 (96.3)
Diarrhoea (%)	297 (99.0)	502 (98.4)	499 (95.0)	642 (96.3)
Blood in stool (%)	295 (98.3)	505 (99.0)	501 (95.4)	642 (96.3)
Fever (%)	297 (99.0)	506 (99.2)	506 (96.4)	640 (96.0)
*Reporting signs/symptoms*				
Blood in urine (%)	51 (17.0)	141 (27.6)	106 (20.2)	117 (17.5)
Painful urination (%)	34 (11.3)	107 (21.0)	132 (25.1)	118 (17.7)
Diarrhoea (%)	33 (11.0)	95 (18.6)	88 (16.8)	93 (13.9)
Blood in stool (%)	50 (16.7)	77 (15.1)	173 (33.0)	155 (23.2)
Swollen abdomen (%)	6 (4.4)	42 (31.1)	42 (31.1)	45 (33.3)
Abdominal pain (%)	74 (24.7)	162 (31.8)	178 (33.9)	154 (23.1)
Fever (%)	143 (47.7)	255 (50.0)	246 (46.9)	244 (36.6)
*Health seeking behaviour (any action), for those reporting signs/symptoms*				
Blood in urine (%)	15 (29.4)	33 (23.4)	50 (47.2)	71 (60.7)
Painful urination (%)	13 (38.2)	34 (31.8)	77 (58.3)	67 (56.8)
Diarrhoea (%)	25 (75.8)	78 (82.1)	77 (87.5)	68 (73.1)
Blood in stool (%)	32 (64.0)	44 (57.1)	126 (72.8)	97 (62.6)
Swollen abdomen (%)	3 (50.0)	18 (42.9)	30 (71.4)	35 (77.8)
Abdominal pain (%)	60 (81.1)	137 (84.6)	135 (75.8)	118 (76.6)
Fever (%)	134 (93.7)	238 (93.3)	229 (93.1)	219 (89.8)
*Health seeking behaviour: visiting health facility (hospital or clinic) as first action for those reporting signs/symptoms*				
Blood in urine (%)	2 (3.9)	7 (5.0)	20 (18.9)	23 (19.7)
Painful urination (%)	4 (11.8)	10 (9.3)	31 (23.5)	24 (20.3)
Diarrhoea (%)	7 (21.2)	10 (10.5)	29 (33.0)	19 (20.4)
Blood in stool (%)	7 (14.0)	3 (3.9)	40 (23.1)	25 (16.1)
Swollen abdomen (%)	0 (0.0)	6 (14.3)	18 (42.9)	11 (24.4)
Abdominal pain (%)	16 (21.6)	18 (11.1)	41 (23.0)	29 (18.8)
Fever (%)	37 (25.9)	45 (17.6)	83 (33.7)	67 (27.5)

*High educational level means attended senior secondary school or tertiary education such as polytechnic and university.

# Three properties car, fridge and television were used as indicators for high socio-economic status.

**†:** The denominators do not include those who said they had not visited a health facility or new entrants who had not yet visited a health facility.

**‡:** Refers to the question what they would do if confronted with the following symptoms. For those aged under six years or older who could not answer the questions themselves, a parent or guardian answered for them.

Many of the 2,002 subjects reported to have experienced schistosomiasis-related symptoms in the past month: 28.4% reported abdominal pain, 22.7% blood in stool, 20.7% blood in urine, 19.5% painful urination, 15.4% diarrhoea and 6.7% swollen abdomen. Fever was reported by 44.4% of the population. All seven symptoms were reported in each study site, although *S. mansoni* is not present in the central study area (Table 1). The proportions of people with these symptoms did not differ markedly between study sites, except for swollen abdomen, which was reported much less in the north (4%) than in the other areas (31–33%). Overall, patients in the south showed higher tendency to take action or visit a health facility for their signs or symptoms than patients from the central or north.

**Figure 1:**
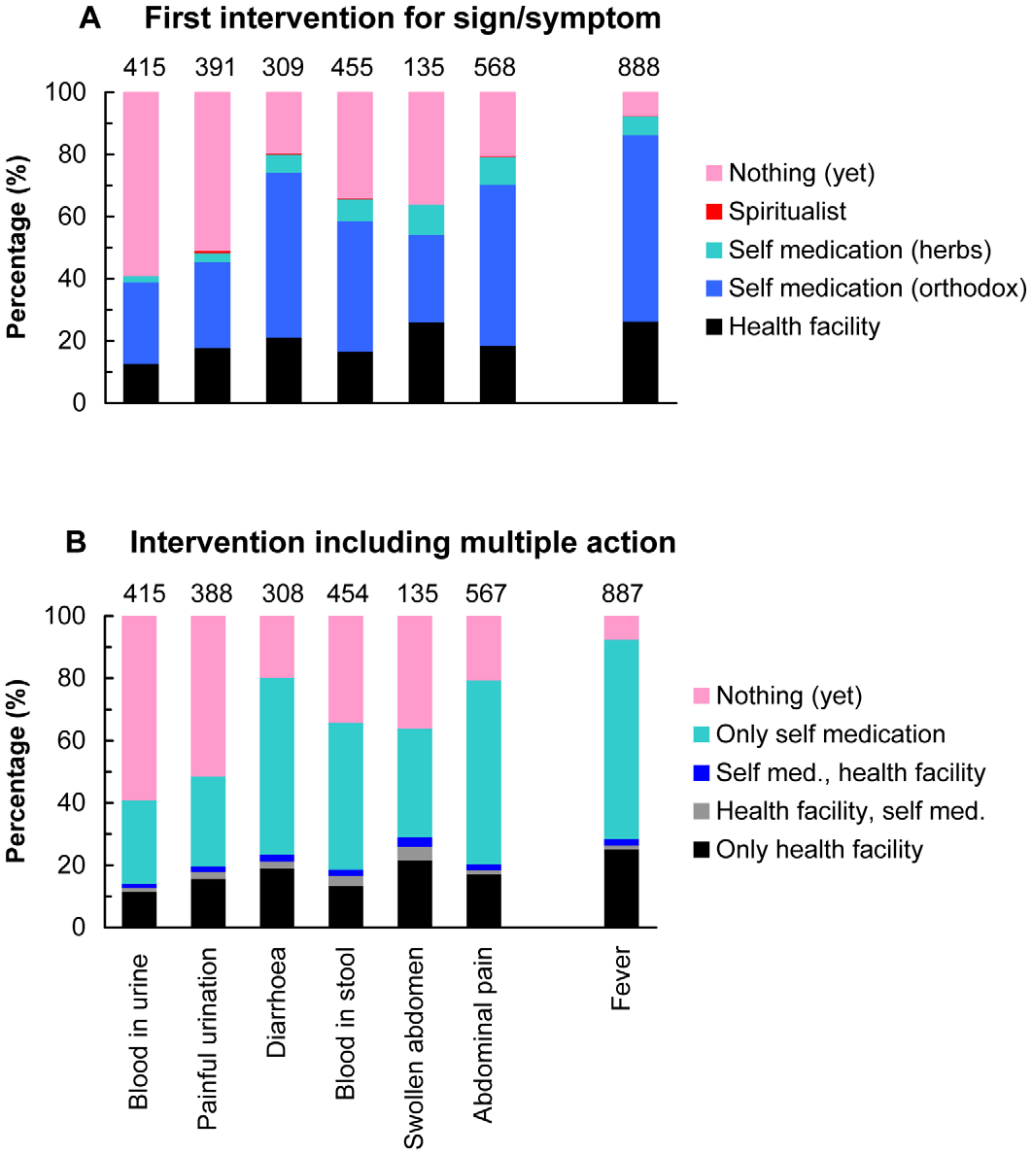
Reported action taken about schistosomiasis-related signs and symptoms. (A) First action only and (B) first and second actions (first action followed by second)**.** The values on top of the bars represent the total number of cases based on 1 month recall period. ‘Health facility’ indicates a hospital, clinic or health centre and spiritualist means traditional healers. Self medication in Figure 1B includes herbal treatment, allopathic medicines, and spiritualist care.

Figure 1A shows main actions taken for schistosomiasis-related symptoms and fever. Doing nothing, self-medicating and visiting a health facility were commonly reported. The large majority of people with blood in urine (60%) or painful urination (>50%) did not seek health care, whereas >90% with fever and about 80% with diarrhea and abdominal pain did take action, mostly self-medication with allopathic drugs. Overall, about 20% schistosomiasis-related symptoms (17% of urinary and 21% of intestinal symptoms) were reported to health facilities as first action, and the percentage did not differ much across symptoms. The proportions visiting a health facility for fever (30%) did not differ appreciably from those visiting with schistosomiasis-related symptoms (20%) (Figure 1B). Multiple actions for a symptom were practiced with some of those self-medicating subsequently visiting a health facility as a second or third alternative and vice versa. The tendency to seek health care was lowest for the symptoms with chronic characteristics (blood in urine, painful urination, swollen abdomen), but when these patients do something, they are more likely to go to the health facility.

People who reported not to have visited a hospital or health centre for their symptoms, were asked for the reasons why not (multiple answers were allowed). Most frequently mentioned reasons were a lack of money (34.5%) and symptom not serious enough (25.3%). More detailed information about determinants of health seeking behaviour was obtained by logistic regression analyses.

The multivariate analysis revealed that people more often took action when they perceived their symptom as severe. This effect was consistent for all the symptoms (Table 2). Age, socio-economic status, someone else providing money for health care showed some effect, but no clear pattern. Sex, duration of symptom, perceived quality of health facility and time for visiting health facility did not demonstrate a significant impact. Usually, symptoms more often led to action for young children (aged 0–9) than for teenagers and to a lesser extent for adults. Although this effect was usually not significant, the direction was fairly consistent. Similarly, the tendency to take action for symptoms is higher for high socio-economic status or if someone other than the respondent paid for health care. The effect of location is less marked, although people in the central region were less inclined to seek health care than in the south, and to a lesser extent north.

**Table 2:** Determinants of taking any health action for schistosomiasis-related symptoms.

Factor	Blood in urine(n = 415)	Painful urination(n = 391)	Diarrhoea(n = 309)	Blood in stool(n = 455)	Swollen abdomen(n = 135)	Abdominal pain(n = 568)	Fever(n = 888)
Age 0–9 vs 20+ years	2.8 (0.5–15)	1.2 (0.4–3.2)	2.2 (0.7–7.5)*	0.84 (0.42–1.7)	0.34 (0.05–2.2)	1.8 (0.8–4.2)*	3.4 (1.5–7.7)**
Age 10–19 vs 20+ years	3.2 (0.6–17)*	0.67 (0.26–1.7)	0.61 (0.19–1.9)	0.36 (0.18–0.73)**	0.56 (0.10–3.1)	1.5 (0.6–3.4)	0.63 (0.35–1.1)*
Female vs male	0.85 (0.41–1.7)			0.81 (0.52–1.3)			0.61 (0.36–1.0)*
SES high vs low	1.1 (0.5–2.4)	2.1 (1.0–4.2)**					1.8 (1.0–3.2)*
North vs South (Manheim)	0.31 (0.09–1.1)*	0.68 (0.21–2.2)	1.0 (0.3–3.3)	1.7 (0.8–3.4)*	(0.0–INF)	0.96 (0.29–3.1)	
Central vs South (Manheim)	0.14 (0.05–0.41)**	0.62 (0.26–1.5)	0.88 (0.33–2.3)	0.91 (0.50–1.7)	0.14 (0.02–1.1)*	0.64 (0.24–1.7)	
South (Tomefa) vs South (Manheim)	0.43 (0.16–1.2)*	1.2 (0.5–2.8)	2.7 (0.8–8.9)*	2.1 (1.2–3.4)**	0.41 (0.05–3.3)	1.0 (0.4–2.7)	
Moderate vs mild	0.81 (0.35–1.8)	1.1 (0.5–2.3)	1.0 (0.4–2.5)	2.0 (1.2–3.2)**	2.0 (0.4–11)	3.5 (1.6–7.9)**	2.3 (1.3–4.0)**
Severe vs mild	3.7 (1.4–9.8)**	1.6 (0.8–3.5)	4.0 (1.2–13)**	3.3 (1.7–6.4)**	6.4 (1.0–42)*	4.5 (1.8–11) **	4.6 (2.2–9.8)**
Acute vs chronic	0.66 (0.17–2.6)			1.5 (0.9–2.4)*	1.3 (0.1–16)		
Other vs self paying for health care	7.6 (1.2–46)**	1.7 (0.6–4.7)	1.7 (0.5–6.1)	1.0 (0.5–2.1)			
QHF somewhat good vs good	1.2 (0.5–2.9)	0.94 (0.43–2.1)	2.6 (0.7–9.7)*			0.64 (0.28–1.5)	
THF less than 2 hours vs ≥ ½ day‡	1.2 (0.5–3.0)	1.4 (0.6–3.4)			1.7 (0.2–15)	1.6 (0.6–4.1)	

The values in the cells are odds ratios (95% CI) from multivariate logistic regression analysis. The empty cells indicate factors resulting in p>0.2 from the univariate analysis and that were hence not included in the multivariate model. INF means infinite, i.e. >999, QHF indicates perceived quality of health facility (hospital) visited in the last instance and THF is time spent visiting the health facility.

*Factors with an effect of 0.05≤ P<0.20.

**Factors with an effect of P<0.05.

**‡:** There were no observations with THF between 2 and 4 hour.

Table 3 shows the determinants of visiting a health facility (hospital or clinic) as first action, in the group of people taking action. Effects are clearest for fever, but are also rather consistent over other symptoms. The direction of the effects is usually the same as described in Table 2, but patterns are more pronounced. Severe symptoms consistently led to an increased tendency to visit a hospital. There were some differences in health seeking behaviour variables across study sites: the people in Tomefa (far from health facility) were more likely to go to the health facility than in Manheim (near), while those in the central region were less likely to do so. Here, duration of symptoms showed effect: patients were less likely to visit the hospital as first action for acute symptoms than for chronic symptoms. The effects of age and socio-economic status were in the same direction as for any action. The other factors tested did not show consistent effects.

**Table 3:** Determinants of health facility (hospital/clinic) utilization for schistosomiasis-related symptoms.

Factor	Blood in urine(n = 52)	Painful urination(n = 69)	Diarrhoea(n = 65)	Blood in stool(n = 75)	Swollen abdomen(n = 35)	Abdominal pain(n = 104)	Fever(n = 232)
Age 0–9 vs 20+ years		0.39 (0.10–1.5)*	3.0 (1.5–6.0)**	2.2 (0.9–5.6)*		1.5 (0.8–3.1)	1.5 (1.0–2.4)*
Age 10–19 vs 20+ years		0.49 (0.15–1.5)	0.97 (0.41–2.3)	2.5 (0.9–6.7)*		1.3 (0.6–2.7)	0.62 (0.36–1.1)*
Female vs male		1.8 (0.6–5.8)					1.4 (1.0–2.0)*
SES high vs low		1.2 (0.5–2.8)		1.7 (0.8–3.9)*		2.1 (1.1–4.1)**	1.5 (1.0–2.1)**
North vs South (Manheim)	0.18 (0.03–0.97)**	0.67 (0.15–3.0)	1.4 (0.5–4.0)	1.7 (0.5–6.7)	(0.0–INF)	1.5 (0.6–4.1)	1.0 (0.6–1.8)
Central vs South (Manheim)	0.16 (0.04–0.65)**	0.70 (0.20–2.4)	0.59 (0.24–1.4)	0.29 (0.07–1.3)*	0.34 (0.11–1.1)*	0.82 (0.33–2.0)	0.61 (0.38–1.0)**
South (Tomefa) vs South (Manheim)	1.0 (0.4–2.9)	1.5 (0.6–3.9)	2.4 (1.2–5.1)**	2.5 (1.0–6.4)**	3.3 (1.1–9.8)**	1.7 (0.8–3.6)*	1.6 (1.0–2.4)**
Moderate vs mild	1.2 (0.4–3.7)	1.5 (0.5–4.3)	1.1 (0.5–2.5)	1.6 (0.6–3.8)		1.0 (0.5–2.1)	1.1 (0.7–1.7)
Severe vs mild	2.1 (0.7–6.6)*	1.6 (0.6–4.3)	2.5 (1.1–5.4)**	3.3 (1.2–9.1)**		2.3 (1.1–4.7)**	3.0 (1.9–4.5)**
Acute vs chronic			0.33 (0.18–0.60)**	0.29 (0.10–0.84)**		0.32 (0.18–0.58)**	0.50 (0.35–0.69)**
Other vs self paying for health care							0.61 (0.37–0.98)**
QHF somewhat good vs good					1.9 (0.6–5.8)		
THF less than 2 hours vs ≥ ½ day‡	1.6 (0.6–4.5)	2.8 (1.1–7.5)**		0.37 (0.14–0.96)**		1.1 (0.5–2.1)	

The values in the cells are odds ratios (95% CI) from multivariate logistic regression analysis (see Table 2 for further explanation).

*Factors with an effect of 0.05≤P<0.20.

**Factors with an effect of P<0.05.

**‡:** There were no observations with THF between 2 and 4 hours.

## Discussion

### Health seeking behaviour and its determinants

This questionnaire-based study was conducted in three regions of Ghana to investigate health seeking behaviour and utilization of health facilities for schistosomiasis-related symptoms. When manifesting schistosomiasis-related symptoms or fever, many people do not take action (50–60% for urinary symptoms, 20–40% for intestinal symptoms, versus <10% for fever) and others only try self-medication (usually with allopathic drugs). Overall, about 20% of all urinary and intestinal schistosomiasis-related symptoms were reported to a health facility, which was lower than the 30% for fever. Strikingly, patients with urinary symptoms show very low tendency to seek care compared to fever and to a lesser extent, intestinal symptoms. The rate of health care seeking is lowest for symptoms with chronic characteristics, but when patients take action, they are slightly more likely to report to a health facility. In a multivariate logistic regression analysis, perceived severity consistently showed to be the most important predictor of both health care seeking in general and visiting a health facility for those that take action.

Our findings are consistent with those from our earlier study [15], although there are some differences in health seeking behaviour variables across the study sites. Generally, people from the south were more likely to take action and visit a health facility than those from central and north. For intestinal schistosomiasis, the difference though may partly be attributed to variation in endemicity between the regions (high in the south, moderate in the north, non-endemic in central), which may be correlated to average worm densities, disease severity and knowledge about the disease. This cannot fully explain differences for urinary schistosomiasis. Other possible explanations include a somewhat higher mean socio-economic status and educational level, improved knowledge and awareness about the disease and its consequences in the south (more education, more schistosomiasis research activities in recent times).

The mechanisms driving health seeking behaviour are complex, requiring multidimensional approaches that bring together all aspects related to access and utilization of health care [19]. Our study confirms the importance of perceived severity of schistosomiasis-related symptoms for health seeking behaviour, as previously reported [20],[21]. We also confirm that people with higher socio-economic status more frequently seek health care and visit health facilities than people with low socio-economic status [12],[22], although the effect of socio-economic status was not always significant. This is also consistent with the frequent reporting of ‘lack of money’ as a reason for not seeking care for symptoms. Our study was not designed to explicitly study the impact of distance, but some information can be obtained by comparing results for the two sites in the south; people from Tomefa have to travel to Mannheim for health care. Whereas earlier studies showed a clear impact of distance on the utilization of health care facilities in rural Nigeria, where utilization declined exponentially with distance [23], we could not confirm this. Contrary to our expectations, people from Tomefa often showed a higher tendency to take any action or to visit the health facility in Mannheim than people from Mannheim itself, after correction for other factors.

Perceived quality of care provided by health facilities did not come out as important determinant. We hypothesized beforehand that lack of adequate diagnosis, care and treatment of patients with schistosomiasis-related symptoms by health facilities could affect utilization. A previous study by our team involving 70 health facilities in four geographical regions in Ghana investigating schistosomiasis case management and means for diagnosis and treatment within the health system demonstrated that symptoms related to *S. haematobium* had a reasonable chance of receiving a correct prescription (60%), compared to <20% for symptoms related to *S. mansoni* [24]. Furthermore, of the health care facilities that would prescribe praziquantel, only 60% had it in stock. Therefore, health professionals in peripheral facilities normally refer patients for diagnostic test and/or treatment [15],[24]). In the current study, patients were asked about perceived quality of health facility visited in the last instance and the results demonstrated an overwhelming majority (>95%) that rated services and care as good (data not shown). Also, poor quality of health facilities was hardly mentioned as a reason for non-use by those not attending hospital. Even though cases of blood in stool and diarrhea have a low chance of receiving adequate diagnosis and treatment, higher proportions were reported to health facilities than blood in urine. These findings all indicate that adequate diagnosis and treatment of schistosomiasis-related cases does not influence service utilization very much.

Inconsistency in the effects of other possible determinants on health seeking behaviour is possibly caused by relatively low numbers of people with specific symptoms. We should also consider the possibility of inaccuracies in the questionnaire-derived data, which can arise from recall bias or social-desirability bias. We do not expect a significant recall bias; we chose a one-month recall period for our interview. Several studies measuring acute and chronic disease properties have shown that for a one-month recall period recall bias is minimal [25],[26],[27]. For schistosomiasis, which presents both acute and chronic characteristics, a careful evaluation concluded that a one-month recall period is appropriate [15], whilst Van der Werf *et al*. [28] found no effect of recall period length for blood in urine due to urinary schistosomiasis. We also don't think that social desirability influenced our results much. Social-desirability might lead to over-reporting of positive practices such as hospital visit. Therefore, for verification, we checked the hospital records that patient keep in their homes. There was good agreement between reported hospital visit and the questionnaire data suggesting that patients did not over-report their visits to a health facility. Further, given the large number of people interviewed and the consistent direction of the responses, it is unlikely that social-desirability may have affected the results. We did not study seasonal variation in health seeking behaviour over the year, but consider it unlikely to have a significant effect, because schistosomiasis transmission and availability of money and time to seek health are more or less stable over the year.

### Implications for control

Our study raises the question whether the observed health seeking behavior is appropriate for symptom relief and preventing progression of the disease in individual patients and morbidity control. These questions are not directly answered by this study. Longitudinal studies or mathematical models for the natural history and treatment effects could help to address this issue. Here, we highlight some factors that are of importance to the prevention of morbidity and sketch policies that can improve the impact of passive case finding or can be implemented as supplementary.

Although the number of cases attending hospital for schistosomiasis-related symptoms (around 20%) is rather low, there are indications that important risk groups are well represented. Firstly, hospital visit is expected to be selective towards those with heavy infections. Hewlett and Cline [29] observed severity of symptoms as an important determinant for passive case reporting to the clinic for urinary schistosomiasis symptoms. A study in a village in Cameroon showed selective hospital visit for haematuria by high intensity cases that formed only 13% of the overall infection in the population [30]. Our data confirm that the vast majority of patients who seek health care or visit a health facility do so when they perceive the symptom as severe. The 20% that visit a health care facility with early symptoms may represent most of those at risk of developing severe long term consequences of the disease. Secondly, the very young children aged <5 years also seem well represented in the treatment group. There is much concern about these young children [31],[32],[33], because schistosomiasis often leads to serious complications like nutritional deficiencies, retarded growth, reduced physical activity, and impaired cognitive function [10],[34]. The finding that children under 10 years showed the highest tendency to visit a hospital/clinic is positive for the potential of passive case finding to prevent morbidity.

The large number of people not visiting a health centre and delay in seeking care are nevertheless of concern. If treatment is not provided early enough, schistosomiasis can lead to long-term serious disease [35]. The development of severe chronic disease is insidious and many late-stage complications are rarely acknowledged to be due to schistosomiasis because there is no clear link between infection earlier in life and later development of severe disease. In itself, it is good news that many people are inclined to seek some form of care for schistosomiasis-like symptoms. However, there seems to be a delay, because many people do not seek care for symptoms that they perceive as mild or moderate and wait until these are severe. Also, when taking action, many people go for self-medication which will not be very effective. Our earlier study revealed that over 90% of those that self-medicated or visited chemical shops for treatment did not receive praziquantel [15]. Also, there is no known traditional medicine for treating schistosomiasis-related symptoms. In many patients, therefore, the symptoms may not be relieved and risk of late morbidity is not or only partly averted.

The case for passive case finding is compelling: most parts of Africa where the highest burden of the disease is concentrated did not benefit from the donor-supported vertical control programmes [9] and although praziquantel use has increased considerably in sub-Saharan Africa following the launch of the ‘Schistosomiasis Control Initiative’ in 2003 [10],[36],[37] the coverage is still limited. The price of PZQ has fallen enormously [38],[39],[40] making it affordable for national health care services to procure the drug and stock it in health facilities. There is a clear potential for control via passive case finding, because a large proportion of people is already taking some action. However, for the reasons explained above, additional measures are probably required. Options include policies to improve quality and accessibility of services health education to stimulate their utilization, or implementation of vertical disease-specific control programmes.

Recent changes in the health care system in Ghana may be positive for integrated control by passive case finding. In the 1990s, the system of payment for health care delivery within the formal sector was out of pocket payment (‘cash and carry’) where a patient was required to make full payment for consultation before treatment was provided, limiting utilization of health services by the poor [41]. However, the Ghana health care delivery system has been undergoing restructuring since the millennium to strengthen the peripheral health facilities where most of these cases are reported. Also a new highly subsidised National Health Insurance Scheme (NHIS) has been introduced after 2005 and adult Ghanaians are to pay a monthly minimum subscription of six thousand Ghanaian Cedis (US $0.66). In this regime of payment, the aged, poor and children of parents who both subscribe to the scheme receive free treatment. The scheme is considered the best alternative to the rigid *cash and carry* that pushed health care far beyond the reach of the poor. The rural poor can now receive medical care for a minimum fee.

Health education may raise awareness in the population about schistosomiasis and schistosomiasis-related symptoms, and encourage symptomatic cases to seek care and more specifically treatment with praziquantel. It should be stressed that treatment in early stages (perhaps not always perceived as severe) can help to prevent late morbidity. Extra attention can be given to infants and pre-school children. However, health education is difficult to sustain in poor and low educated rural communities that have to deal with so many other infections at the same time [42],[43]. Health education for schistosomiasis becomes even more complicated as the link between infection earlier in life and late complications is hard to establish. It is worthwhile exploring to what extent passive case finding is enhanced by the changes in health care system and health education.

We cannot rule out that specific disease control programmes will be necessary to achieve more complete morbidity control. Such programmes are sometimes met with criticism because they would draw resources from the general health system and perhaps not viable in the long run, but they can be very effective. One option is to target chemotherapy at school-aged children and adolescents, as these age groups show the highest intensities of infection. This has led to school health programmes, which have demonstrated promising results [44],[45],[46]. A setback is that in rural settings of most African countries many children do not attend school and are not reached by such programmes. Community-directed mass treatment represents another possibility, which has successfully been used for the control of diseases such as lymphatic filariasis and onchocerciasis, and is currently being tried for schistosomiasis [46],[47]. There are a number of operational problems including drug procurement, but the strategy is well-accepted by communities as local members are fully involved and take responsibility of delivery of treatment [48]. When passive case finding is supplemented with one or more of these control efforts, it may shorten the delay in getting treatment, improve coverage and decrease rates of self-medication for people suffering from schistosomiasis.

### Concluding remarks

Perceived severity of the disease is the most important determinant for visiting a hospital or clinic in Ghana for schistosomiasis-related symptoms. The role of other factors was less clear and sometimes inconsistent. Additional studies are required to further develop and fully understand the multidimensional framework that explains how access and utilization of health services depend on characteristics of a symptom, individual, household, community, health facility, health systems, etc. Schistosomiasis control by passive case-finding within the regular health care delivery looks promising, but the number not visiting a health facility is large and calls for supplementary control options.
